# Catheter Ablation of Fascicular Ventricular Tachycardia

**Published:** 2008-08-01

**Authors:** B Ramprakash, S Jaishankar, Hygriv B Rao, C Narasimhan

**Affiliations:** CARE Hospitals and CARE Foundation, Exhibition Road, Nampally, Hyderabad, India. PIN: 500001

**Keywords:** Fascicular ventricular tachycardia, Radiofrequency ablation

## Abstract

Fascicular ventricular tachycardia (VT) is an idiopathic VT with right bundle branch block morphology and left-axis deviation occuring predominantly in young males. Fascicular tachycardia has been classified into three subtypes namely, left posterior fascicular VT, left anterior fascicular VT and upper septal fascicular VT. The mechanism of this tachycardia is believed to be localized reentry close to the fascicle of the left bundle branch. The reentrant circuit is composed of a verapamil sensitive zone, activated antegradely during tachycardia and the fast conduction Purkinje fibers activated retrogradely  during tachycardia recorded as the pre Purkinje and the Purkinje potentials respectively. Catheter ablation is the preferred choice of therapy in patients with fascicular VT. Ablation is carried out during tachycardia, using conventional mapping techniques in majority of the patients, while three dimensional mapping and sinus rhythm ablation is reserved for patients with nonmappable tachycardia.

## Introduction

The first report of fascicular ventricular tachycardia was in 1979 by Zipes et al [1]; they described a ventricular tachycardia (VT) of right bundle branch block morphology with left-axis deviation and a relatively narrow QRS width. Belhassen et al [2] in 1981 observed that intravenous administration of verapamil significantly reduced the rate of this VT and prevented its subsequent reinitiation during electrphysiological study. At present this tachycardia is well characterized as one presenting as exercise-related VT in the age group of 15 to 40 years and predominantly in males (60% to 80%). Fascicular VT occurs in patients with no structural cardiac abnormality and is usually paroxysmal, but it can occasionally be incessant in nature resulting in tachycardiomyopathy [3]. While termination by calcium channel blockers is the hallmark of this tachycardia, catheter ablation is very effective in the cure of this tachycardia. This review summarizes the current concepts of the mechanism, electrophysiologic properties and various method of catheter ablation of this tachycardia.

## Anatomic and physiologic properties

Zipes et al [3] postulated that the relatively narrow morphology of the QRS during the arrhythmia localized the origin of the arrhythmia close to the posterior fascicle of the left bundle branch. Anatomically, there is speculation that the tachycardia might originate from a false tendon or fibromuscular band that extends from the posteroinferior left ventricle to the basal septum [4,5]. Studies by Ouyang et al by CARTO mapping suggest that the electroanatomic substrate for fascicular VT may be indicative of involvement of  the posterior Purkinje network [6].

**Figure 1:**
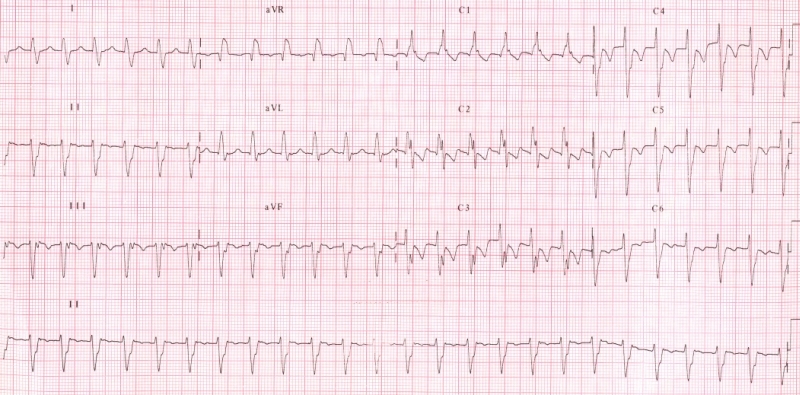
Surface 12 lead ECG during fascicular VT showing a right bundle branch block QRS morphology with left axis deviation, QRS duration is of 120 ms.

Fascicular tachycardia has been classified into three subtypes [7] (1) left posterior fascicular VT with a right bundle branch block (RBBB) pattern and left axis deviation (Figure 1) (2) left anterior fascicular VT with RBBB pattern and right-axis deviation and (3) upper septal fascicular VT with a narrow QRS and normal axis configuration. Upper septal fascicular tachycardia usually exhibits right bundle branch block morphology. Rare cases exhibiting LBBB (precordial R wave transition between V3 and V4) and a normal frontal plane axis have been reported [8]. The commonest form of fascicular tachycardia is the posterior fascicular type accounting for nearly 90% of the cases [9].

## Electrophysiological characteristics

Fascicular tachycardia can be induced by programmed atrial or ventricular stimulation. Administration of isoprenaline by infusion may be needed to facilitate induction in some cases; however this tachyarrhythmia may be non inducible in a significant number of patients [10,11]. It is important to note that 10 to 25% of patients with idiopathic fascicular tachycardia also have an associated supraventricular tachycardia inducible during programmed stimulation [11,12].

### Tachycardia circuit

**Figure 2:**
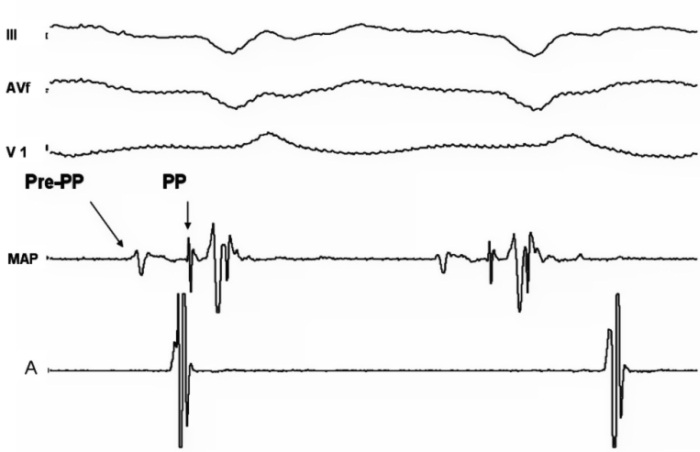
Surface and intracardiac recording during fascicular VT with the MAP catheter in the left ventricle against the apical septum and atrial catheter in the high right atrium showing atrioventricular dissociation. The pre Purkinje potential (Pre-PP) is seen as the first deflection in the MAP catheter, it is  a comparatively dull, lower frequency potential, followed by the Purkinje potential (PP) which is sharp, high frequency potential, preceding the onset of QRS during tachycardia.

The tachycardia circuit can be constructed from the observations during VT. During tachycardia two distinct potentials can be observed before the ventricular electrogram, namely the purkinje potential (PP) and pre Purkinje potential (Pre-PP), also designated P2 and P1 respectively (Figure 2) [13,14]. The relative activation times of PP and pre-PP to the onset of QRS complex vary between   5-25 ms and 30-70 ms respectively.  The Purkinje potential (PP or P2) first described by Nagakawa et al represents the activation of the left posterior fascicle or the Purkinje fiber near the left posterior fascicle. It is characterized by a brief, sharp, high-frequency potential preceding the onset of QRS during tachycardia [15]. The pre Purkinje potential (Pre-PP or P1) described first by Tsuchiya et al represents excitation at the entrance to the specialized zone in the ventricular septum which has decremental properties and is sensitive to verapamil [16]. The pre-PP is a comparatively dull, lower frequency potential preceding the PP during tachycardia. This area is captured antidromically during tachycardia and at higher pacing rates; thereby the pre-PP precedes the PP during tachycardia. Whereas it is captured orthodromically in sinus rhythm and at relatively lower pacing rates, hence the pre PP follows the ventricular complex. The close association of fast conducting Purkinje fibers and the slow conducting verapamil sensitive fibers set the stage for a reentrant mechanism.

**Figure 3:**
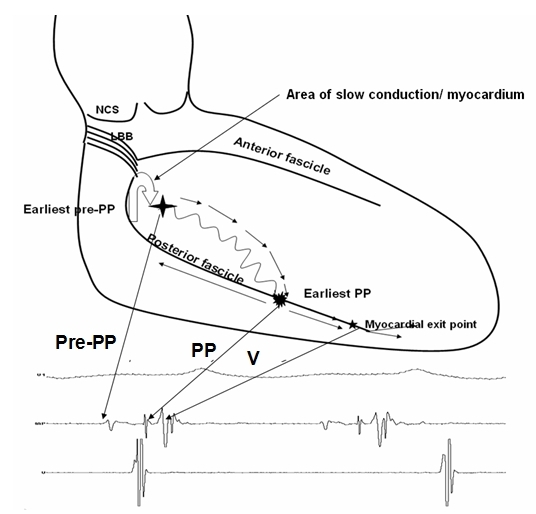
Diagrammatic representation of the tachycardia circuit in fascicular VT. The antegrade limb of the circuit proceeds through the verapamil sensitive zone (curved line) from basal to apical left ventricular septum giving rise to the Pre PP as seen in the accompanying electrogram. The lower turn around site of the reentrant circuit occurs in the lower third of the septum with the capture of the fast conduction Purkinje fibers along the posterior fascicle. From here, antegrade activation occurs down the septum to break through septal myocardium below, and retrograde activation occurs over the posterior fascicle from apical to basal septum forming the retrograde limb of the tachycardia. The reentrant circuit is completed by a zone of slow conduction at the upper turn around point of the circuit located close to the main trunk of the left bundle branch.

During VT, the antegrade limb of the circuit proceeds through the specialized, verapamil sensitive zone from the basal to the apical site of the left ventricular septum, giving rise to the Pre PP. Therefore the earliest Pre-PP is seen in the basal septum and the latest pre-PP is seen in the apical septum. The lower turn around site of the reentrant circuit occurs in the lower third of the septum with the capture of the fast conduction Purkinje fibers which are rapidly activated in either direction during tachycardia. Antegrade activation occurs down the septum to break through in the posterior septal myocardium below, and retrograde activation occurs over the posterior fascicle from apical to basal septum forming the retrograde limb of the tachycardia. The reentrant circuit of fascicular tachycardia is completed by a zone of slow conduction between Pre PP and PP areas in the basal interventricular septum. The slow conduction zone which is the upper turn around point of the circuit is located close to the main trunk of the left bundle branch (Figure 3).  The opposite wavefronts in the two limbs can be recorded as opposite activation sequence of pre-PP and PP during the tachycardia, with PP occurring earliest and pre-PP occurring latest in the lower septum and widely separate pre-PP and PP in upper septum [14].

## Differential diagnosis

**Figure 4:**
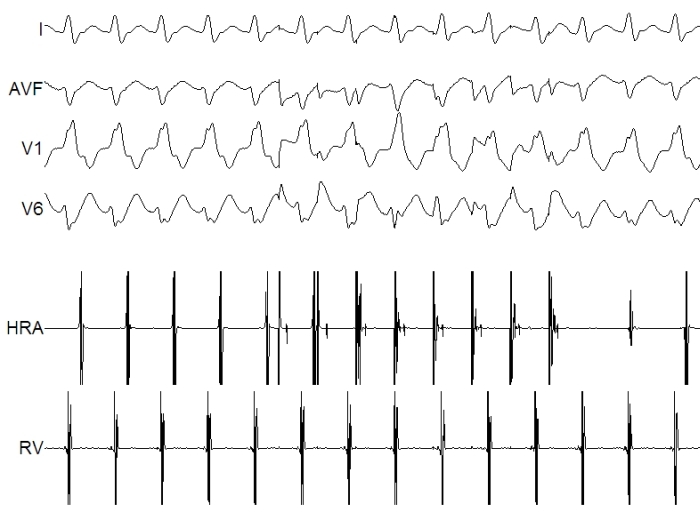
Surface and intracardiac recording during tachycardia showing 1:1 VA association. Atrial pacing performed from high right atrium at a faster rate than the tachycardia clearly dissociates the ventricular activity from atrial activity suggesting the diagnosis of fascicular VT.

Fascicular VT is frequently misdiagnosed as supraventricular tachycardia with aberrancy on account of the narrow-QRS duration during tachycardia (120 ms), responsiveness to intravenous verapamil, and the occurrence in young patients with structurally normal hearts [17]. However a careful analysis of the surface ECG can demonstrate VA dissociation - but in a young patient with robust VA conduction this may not be evident. During EP study, induction and entrainment is possible by atrial stimulation leading to mistaken diagnosis of SVT. However rapid atrial pacing during tachycardia can demonstrate AV dissociation and favors the diagnosis of fascicular VT (Figure 4).

Interfascicular VT, which is a type of bundle branch reentrant VT, has a typical RBBB morphology and left or right axis deviation during VT mimicking fascicular VT. However, interfascicular VT is most commonly seen in patients with an anterior infarction and either left anterior or posterior hemi fascicular block. During EP study, the ventricular depolarization is preceded by His bundle depolarization in interfascicular VT which is not seen in fascicular VT [18].

Idiopathic mitral annulus VT has RBBB morphology with right axis deviation of the frontal QRS vector and hence mimics anterior fascicular VT. Tada et al [19] described presystolic potential preceding QRS complex during arrhythmia in one third of their patients with mitral annulus VT. Some of their cases were inducible by programmed stimulation suggesting a reentrant mechanism and in two patients the VT was terminated by verapamil which is characteristic of fascicular VT. Further definition of this rare tachycardia is needed to clearly differentiate it from fascicular VT.

## Radiofrequency Ablation

The first report of ablation of fascicular VT was in 1987 by Fontaine et al [20] by the application of a high energy DC shock  in the inferoseptal area. Subsequently, Klein et a1 [21] described successful treatment by radiofrequency current in one patient with idiopathic left ventricular tachycardia. Since then various methods of mapping and targets for ablation during VT and during sinus rhythm have been described.

### Ablation during tachycardia

Usually ablation of this tachycardia is performed during VT because the electrophysiologic targets are clear and the end point of ablation is termination of tachycardia. Various investigators have used different targets for ablation during tachycardia.

#### Ablation at Purkinje potential site

Nakagawa et al [15] preferred careful localization of the PP in guiding ablation. A careful search is performed for the PP preceding the onset of QRS during tachycardia in an area over 2 to 3 square centimeters in the posterior half of the left ventricular septum, one quarter to one third of the distance from apex to base. Radiofrequency energy is delivered at this site recording the earliest PP considered to be the lower turn around point of the reentrant circuit. This area is located more basally than the left ventricular area that shows earliest ventricular activation during tachycardia (the exit point of the circuit into ventricular septum). Therefore the location of the earliest ventricular activation during tachycardia is not an ideal site for ablation of this arrhythmia. Nogami et al [14] observed that the interval between PP at the site of successful ablation and the onset of the QRS complex during VT was 18 ± 6 ms (6 ±3% of VT cycle length). Apart from localization of the target site by earliest PP, entrainment can be performed from the target site to assess the likelihood of success. Pacing from the successful ablation site produces entrainment with concealed fusion, and post pacing interval minus VT cycle length difference within 30 ms. During pacing from the distal PP recording site, a stimulus to QRS interval same as PP to QRS interval during VT predicts success during ablation [13].

#### Ablation at pre Purkinje potential site

Aiba et al [13] and Nogami et al [14] targeted the pre-PP during VT. The pre-PP is recordable in three fourth of the patients during VT. [4] It is recorded within a small area proximal to the earliest PP recording site, and is activated from the basal to apical septum toward the earliest PP site. The interval between Pre-PP at the site of successful ablation and the onset of the QRS complex during VT was 60 ± 29 ms (18 ± 8% of VT cycle length).  After successful ablation, pre PP appears after the QRS complex during sinus rhythm. However, performance of ablation at this site carries the risk of causing atrioventricular block or left bundle branch block. Arya et al compared retrospectively the significance of recording PP or pre-PP in the selection of ablation target and number of RF application. Fewer applications (4.7 ± 1.8) were needed in whom RF ablation was initially targeted to PP compared to those targeted to pre-PP recording site (12.2 ± 3.3, P < 0.05). [22]

### Ablation during sinus rhythm

In patients with non-inducible tachycardia and in those with ill sustained tachycardia during the study, mapping and ablation during tachycardia is not possible. Moreover, the critical substrate of the tachycardia is amenable to mechanical injury due to catheter manipulation, thus making the tachycardia non-inducible during the study. Various methods of mapping and ablation of fascicular VT in such patients have been described.

#### Pace mapping

In general, pace mapping is more useful in mapping focal tachycardias whereas outcomes in reentrant tachycardia ablation are less favourable [23,24]. In the ablation of fascicular VT, though pace mapping may be a useful guide to the site for ablation, a perfect pace map is not essential for success [15,25]. Nogami et al [14], demonstrated that pacing from successful site of ablation showed a match in the VT morphology only in  9.6 ± 2.1 of the 12 ECG leads. This may be due to the capture of pathways within the Purkinje network that are not included in the reentry circuit and/or adjacent myocardium. However, Wellens et al [13] advocated pace mapping for localizing the site of ablation.

#### Electro anatomic mapping

**Figure 5:**
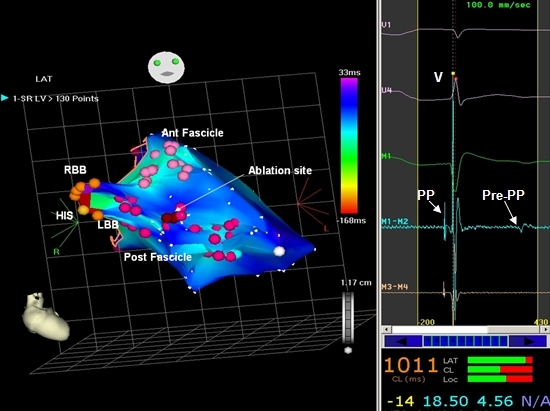
CARTO map of the left ventricle during sinus rhythm in a patient with fascicular VT. The conduction system is mapped and tagged from the HIS bundle to the peripheral insertion of the fascicles. Ablation points are marked in red and the corresponding electrogram is displayed in the inset (M1-M2, Map catheter), showing a sharp Purkinje potential (PP) before the ventricular electrogram and a blunt pre Purkinje potential (pre-PP) after the ventricular electrogram.

Anatomic data gathered together with endocardial electrogram data by various electroanatomic mapping systems have facilitated ablation of this arrhythmia in sinus rhythm. Chen et al [26] reported the use of EnSite 3000 System to create a three-dimensional endocardial geometry of the left ventricle (LV) and the conduction system in the LV during sinus rhythm. The His bundle area, left bundle branch, fascicles and sinus breakout point were mapped in detail and tagged as special landmarks in the geometry. A linear lesion was placed perpendicular to the wave front propagation direction of the left posterior fascicle, 1 cm above the sinus breakout point. They described a valuable end point for this anatomic ablation in that the 12-lead ECG after the linear lesion has been created shows a significant shift of the QRS axis to the right, deep Q waves in inferior leads and deep S waves in lateral leads without the morphology of real left posterior fascicular block. Similarly Lin et al, [10] used CARTO electroanatomic mapping and created a linear lesion midway between the apex and base of the septum, with Purkinje potentials as an additional guide. Ouyang et al [6] used CARTO map for anatomical localization and tagging areas having pre PP, and performed ablation during sinus rhythm at these sites (Figure 5).

## Conclusion

Catheter ablation is the preferred choice of therapy in patients with fascicular VT. The success rate for ablation is more than 80% and complications are infrequent. Most of the patients with fascicular VT can ablated during tachycardia, using conventional mapping techniques. Three dimensional electro anatomical mapping and sinus rhythm ablation is to be reserved for patients with non - inducible or illsustained tachycardia and in patients with recurrences.
